# In vivo plug-and-play: a modular multi-enzyme single-cell catalyst for the asymmetric amination of ketoacids and ketones

**DOI:** 10.1186/s12934-017-0750-5

**Published:** 2017-07-28

**Authors:** Judith E. Farnberger, Elisabeth Lorenz, Nina Richter, Volker F. Wendisch, Wolfgang Kroutil

**Affiliations:** 1 Austrian Centre of Industrial Biotechnology, ACIB GmbH, c/o University of Graz, Heinrichstrasse 28, 8010 Graz, Austria; 2 Genetics of Prokaryotes, Faculty of Biology & CeBiTec, Bielefeld University, 33501 Bielefeld, Germany; 3 Institute of Chemistry, University of Graz, NAWI Graz, BioTechMed Graz, Heinrichstrasse 28, 8010 Graz, Austria

**Keywords:** Biocatalysis, Single-cell biotransformation, *Escherichia coli*, Asymmetric reductive amination, Transaminases, Chiral amines, α-Amino acids, Modular concept, Flexibility

## Abstract

**Background:**

Transaminases have become a key tool in biocatalysis to introduce the amine functionality into a range of molecules like prochiral α-ketoacids and ketones. However, due to the necessity of shifting the equilibrium towards the product side (depending on the amine donor) an efficient amination system may require three enzymes. So far, this well-established transformation has mainly been performed in vitro by assembling all biocatalysts individually, which comes along with elaborate and costly preparation steps. We present the design and characterization of a flexible approach enabling a quick set-up of single-cell biocatalysts producing the desired enzymes. By choosing an appropriate co-expression strategy, a modular system was obtained, allowing for flexible plug-and-play combination of enzymes chosen from the toolbox of available transaminases and/or recycling enzymes tailored for the desired application.

**Results:**

By using a two-plasmid strategy for the recycling enzyme and the transaminase together with chromosomal integration of an amino acid dehydrogenase, two enzyme modules could individually be selected and combined with specifically tailored *E. coli* strains. Various plug-and-play combinations of the enzymes led to the construction of a series of single-cell catalysts suitable for the amination of various types of substrates. On the one hand the fermentative amination of α-ketoacids coupled both with metabolic and non-metabolic cofactor regeneration was studied, giving access to the corresponding α-amino acids in up to 96% conversion. On the other hand, biocatalysts were employed in a non-metabolic, “in vitro-type” asymmetric reductive amination of the prochiral ketone 4-phenyl-2-butanone, yielding the amine in good conversion (77%) and excellent stereoselectivity (*ee* = 98%).

**Conclusions:**

The described modularized concept enables the construction of tailored single-cell catalysts which provide all required enzymes for asymmetric reductive amination in a flexible fashion, representing a more efficient approach for the production of chiral amines and amino acids.

**Electronic supplementary material:**

The online version of this article (doi:10.1186/s12934-017-0750-5) contains supplementary material, which is available to authorized users.

## Background

Enantiopure α-amino acids [1, 2] and amines [3, 4] represent classes of chiral chemicals with versatile application in chiral pharmaceutical and asymmetric synthesis. Biocatalysis [5] has contributed remarkably to the development of economically feasible and sustainable methods for the preparation of these compounds: examples of enzymes comprise imine reductases [6–8], monoamine oxidases [9, 10], amine dehydrogenases [11–14], hydrolytic enzymes [15, 16] or transaminases (TAs) [17–19] for asymmetric functionalization of keto groups. The latter are pyridoxal-phosphate dependent enzymes catalyzing simplified the amino group transfer from a donor amine to a keto group to produce a chiral amine with a new stereocenter and thus a molecule with increased value. An efficient TA-catalyzed amination depends on the removal or recycling of the co-product to shift the reaction equilibrium towards the product side. A range of well-working techniques has been developed like the coupling of l-alanine dependent TAs with an alanine dehydrogenase (AlaDH) and an enzyme for nicotinamide cofactor regeneration, which can be for instance formate dehydrogenase or glucose dehydrogenase [20, 21]. This orthogonal three-enzyme cascade has successfully demonstrated its usefulness in a range of TA-catalyzed transformations and hence has occupied a permanent spot in cell-free biocatalysis involving TAs. Nevertheless, the assembly of multiple biocatalysts in vitro, which is generally termed “systems biocatalysis” [22–24], implies the individual production of each required enzyme, which is time-consuming and lacks elegance. Consequently, considerable effort has been dedicated to the development of microbial cell factories, which are designed to perform multistep biotransformations in vivo. However, instead of exploiting native or engineered metabolic pathways [25, 26], focus has lately been put on the introduction of whole artificial de novo pathways [27–30]. This approach leads to tailored single-cell biocatalysts, which excel related cell-free systems by offering a more inexpensive and easy catalyst preparation and a simplified overall process configuration. Required cofactors can be provided and/or regenerated by the cell using an internal cofactor recycling system coupled to the host’s metabolism. Furthermore, the close proximity of biocatalysts within the confined space of one cell enables consecutive or even concurrent reaction steps in a highly efficient way [31]. Such in vivo cascades have been successfully developed for a variety of useful applications and are highlighted as well as opposed to in vitro approaches in several recent in-depth reviews [32–35]. While at the beginning mostly redox reactions including dehydrogenases, reductases and monooxygenases were considered for tailor-made designer cells [36–39], recently also TA-catalyzed reductive amination attracted attention to being included in artificial in vivo pathway construction [40, 41]. In this context especially the amino functionalization of alcohols by combining an alcohol dehydrogenase (ADH) with a TA in a redox self-sufficient fashion has been extensively studied [42, 43]. Moreover, transamination was investigated with recombinant yeast single-cells of *Saccharomyces cerevisiae*, exploiting cell metabolism for cofactor regeneration [44, 45]. To our knowledge however, there are no reports on transferring the classic TA-catalyzed reductive amination machinery into a single recombinant *E. coli* cell. Therefore, in the present study the orthogonal cascade was translated from an in vitro to an in vivo mediated approach by generating all required enzymes within a bacterial single-cell system. In order to allow for a flexible interplay of multiple enzyme-catalyzed reactions within one cell, thorough catalyst design as well as a careful choice of co-expression strategy were crucial parameters. The microbial cell factory contained three basic enzyme modules, each of them catalyzing one of the reactions constituting the targeted orthogonal cascade: (1) asymmetric reductive amination, (2) amine donor regeneration and (3) nicotinamide cofactor regeneration. Each module offered various enzyme options, which were combined in a plug-and-play fashion for various demands, enabling broad applicability. The obtained toolbox of single-cell biocatalysts was successfully employed for the asymmetric reductive amination of α-ketoacids and prochiral ketones, investigating both fermentative transformations coupled to the host’s metabolism and non-metabolic “in vitro-type” transformations.

## Results

### Design and construction of modular plug-and-play single-cell biocatalysts

**Fig. 1:**
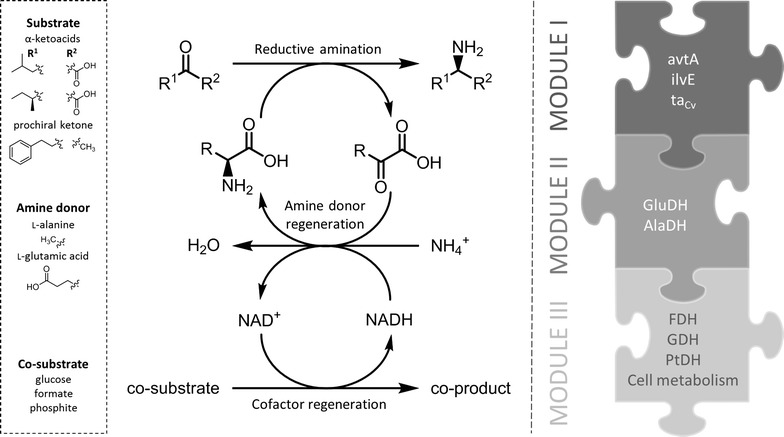
Tailored construction of single-cell catalysts for asymmetric amination using a module-based catalyst design. Module I provides various enzymes catalyzing reductive amination of the target substrate, module II offers two enzymatic options for amine donor regeneration and finally, nicotinamide cofactor recycling is performed by components of module III. The flexible nature of the approach allows for easy substitution of individual enzymes according to the wanted application

**Fig. 2:**
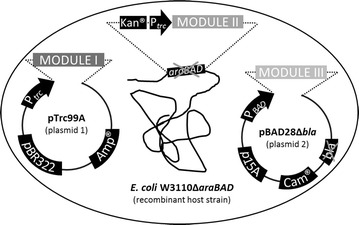
Cloning and co-expression strategy. A recombinant *E. coli* W3110 cell provides enzyme modules I and III on two co-transformed plasmids and enzyme module II integrated into the genome

The aim was the development of a recombinant *E. coli* single-cell system providing all required enzymes for the asymmetric reductive amination of prochiral α-ketoacids and ketones in a flexible plug-and-play fashion. The system consisted of three modules, each of them representing one essential reaction type of the amination system, namely (i) the amination of the target substrate (module I), (ii) the recycling of the amine donor molecule (module II) and (iii) the recycling of the reduced nicotinamide cofactor (module III) (Fig. 1). For each module a library of different constructs encoding specific enzymes was available. Upon combination of selected compatible constructs from each module a single bacterial cell was generated, capable of catalyzing the targeted orthogonal cascade reaction. For module I in our representative study one out of four alternative constructs encoding enzymes for the reductive amination of keto-functionalities can be chosen: a l-alanine–valine aminotransferase (encoded by *avtA*), a branched-chain amino acid aminotransferase either from *E. coli* MG1655 (encoded by *ilvE*_*Ec*_) or *Streptococcus mutans* (encoded by *ilvE*_*Sm*_) for the production of various α-amino acids or a (*S*)-selective transaminase from *Chromobacterium violaceum* [46] (encoded by *ta*_*Cv*_), giving access to a broad range of optically pure amines. The required enzymes for regenerating the consumed amine donor are supplied by module II. While AvtA and Ta_Cv_ rely on l-alanine as amine donor, IlvE is consuming l-glutamate. Consequently, an l-alanine dehydrogenase (AlaDH) and a l-glutamate dehydrogenase (GluDH) from *Bacillus subtilis* were determined as the enzymes of module II. The third module finally provided various options for regenerating the nicotinamide cofactor NADH required for the oxidation reaction catalyzed by AlaDH and GluDH. NADH can either be regenerated in the traditional way by cellular metabolism, using a co-substrate like glucose, or by an enzyme-coupled approach. For the latter option, a toolbox was generated consisting of three constructs encoding either a formate dehydrogenase (FDH) from *Komagataella pastoris* GS115, a glucose dehydrogenase (GDH) from *Bacillus megaterium* and a phosphite dehydrogenase (PtDH) from *Pseudomonas stutzeri*. In order to enable a flexible catalyst design allowing for easy plug-and-play-like combination of desired enzymes, the corresponding genes had to be controlled separately from each other within one cell. Therefore, co-expression of the enzymes required for modules I–III was set-up by combining a two-plasmid strategy with the integration of one gene into the chromosome of the used *E. coli* host (Fig. 2). Genes encoding transaminases were individually cloned into the IPTG-inducible *E. coli* expression vector pTrc99A (plasmid 1) under the control of the P*trc* promoter and ribosome binding sites upstream of each gene (pTrc99A-*avtA*, pTrc99A-*ilvE*, pTrc99A-*ta*_*Cv*_). Genes coding for AlaDH and GluDH were individually integrated into the genome of the used *E. coli* strain by homologous recombination resulting in two hosts. For this purpose, the *araBAD* genes in the chromosome of *E. coli* W3110 were replaced with heterologous *ald* (AlaDH) or *rocG* (GluDH) gene, respectively under the control of IPTG-inducible strong P*trc* promoter [47] and ribosome binding sites upstream of each gene. The new strains were named *Ec*-AlaDH (*E. coli* W3110*ΔaraBAD*::P*trc*-*ald*) and *Ec*-GluDH (*E. coli* W3110*ΔaraBAD*::P*trc*-*rocG*) according to the amino acid dehydrogenase they provided. The genes encoding NADH-recycling enzymes were cloned into pBAD28 (plasmid 2) under control of the arabinose-inducible the P*BAD* promoter [48] and ribosome binding sites upstream of each gene. Due to a different origin of replication (p15Aori) and a chloramphenicol resistance, pBAD28 provides the appropriate features to be compatible with pTrc99A (pBR322ori, *bla*). However, since it also contains the same β-lactamase gene, we removed parts of it in order to inactivate the ampicillin resistance (pBAD28∆*bla*). The newly obtained plasmids were designated pBAD28∆*bla*-*fdh*, pBAD28∆*bla*-*gdh*, pBAD28∆*bla*-*ptdh*.

By co-transformation of the specific recombinant strains with the required compatible plasmid(s), the different enzyme modules were assembled flexibly within one cell and gave rise to a range of single-cell catalysts useful for various applications (Table 1). Due to the fact that transaminase IlvE depends on l-glutamate as amine donor, plasmid pTrc99A-*ilvE* was transformed into strain *Ec*-GluDH. In a first approach the NADH recycling depended on cell catabolism (Table 1, catalyst 1**a** and **b**). The other transaminases AvtA and Ta_Cv_ rely on l-alanine as amino donor and were thus transformed into strain *Ec*-AlaDH.

**Table 1:** Constructed single-cell biocatalysts by flexible in vivo assembly of three modules

Catalyst	*E. coli* strain	Plasmid encoding Ta	Cofactor recycling	Model product
**1a**	*Ec*-GluDH	pTrc99A-*ilvE*_*Ec*_	Cell metabolism	l-Leucine
**1b**	*Ec*-GluDH	pTrc99A-*ilvE*_*Sm*_	Cell metabolism	l-Leucine
**2a**	*Ec*-AlaDH	pTrc99A-*avtA*	Cell metabolism	l-Isoleucine
**2b**	*Ec*-AlaDH	pTrc99A-*avtA*-*ald*	Cell metabolism	l-Isoleucine
**2c**	*Ec*-AlaDH	pTrc99A-*ald*-*avtA*	Cell metabolism	l-Isoleucine
**3a**	*Ec*-AlaDH	pTrc99A-*avtA*	pBAD28Δ*bla*-*fdh*	l-Isoleucine
**3b**	*Ec*-AlaDH	pTrc99A-*ald*-*avtA*	pBAD28Δ*bla*-*fdh*	l-Isoleucine
**4a**	*Ec*-AlaDH	pTrc99A-*avtA*	pBAD28Δ*bla*-*gdh*	l-Isoleucine
**4b**	*Ec*-AlaDH	pTrc99A-*ald*-*avtA*	pBAD28Δ*bla*-*gdh*	l-Isoleucine
**5a**	*Ec*-AlaDH	pTrc99A-*avtA*	pBAD28Δ*bla*-*ptdh*	l-Isoleucine
**5b**	*Ec*-AlaDH	pTrc99A-*ald*-*avtA*	pBAD28Δ*bla*-*ptdh*	l-Isoleucine
**6**	*Ec*-AlaDH	pTrc99A-*ta*_*Cv*_	pBAD28Δ*bla*-*fdh*	4-Phenyl-2-butylamine
**7**	*Ec*-AlaDH	pTrc99A-*ta*_*Cv*_	pBAD28Δ*bla*-*gdh*	4-Phenyl-2-butylamine
**8**	*Ec*-AlaDH	pTrc99A-*ta*_*Cv*_	pBAD28Δ*bla*-*ptdh*	4-Phenyl-2-butylamine

While AvtA was coupled both with glucose catabolism (catalyst 2) as well as with a regenerating enzyme for nicotinamide recycling (catalysts 3–5), the (*S*)-selective transaminase Ta_Cv_ was just used in combination with the enzyme-coupled approach (catalysts 6–8). With the constructed single-cell biocatalysts in hand, optimization of co-expression of multiple enzymes within one cell was required. In order to validate the production of active proteins and an ideal ratio of enzyme activities, we performed photometric activity assays individually for each enzyme, ensuring that all the genes were functionally expressed. Depending on the expression conditions used, crude extracts displayed specific enzyme activities of approximately 0.1–0.3 U mg^−1^ for transaminase Ta_Cv_ and IlvE, whereas a significantly higher transaminase activity of 0.7–1 U mg^−1^ was obtained for AvtA. While GluDH expressed from genome-encoded *rocG* indicated a specific activity of 0.4 U mg^−1^, for AlaDH expressed from the genome-encoded *ald* widely differing specific activities in the range of 0.12–0.36 U mg^−1^ were obtained. In order to increase the amount of AlaDH present in the cell and consequently also its activity, a second *ald* gene copy was introduced to the system via plasmid pTrc99A-*avtA*-*ald*, which has already been constructed previously [49]. Additionally, another vector with a different gene order was constructed in this study, where AlaDH was encoded in the first position of the artificial operon with the P*trc* promoter upstream of *ald* (pTrc99A-*ald*-*avtA*). Indeed, activity assays showed that AlaDH activity was enhanced manifold to 2.1 U mg^−1^ with the latter plasmid. In comparison, crude extracts of the strain DH5α carrying pTrc99A-*avtA*-*ald* displayed only 0.3 U mg^−1^. This result showed that not only the increased number of plasmid-encoded *ald* genes, but especially the distance between promoter and gene in the operon significantly affected expression efficiency (operon polarity). Nevertheless, both plasmid-based gene orders of the artificial *avtA*-*ald* and *ald*-*avtA* operons, respectively were used for further experiments and compared to the genome-based approach. With respect to the third module for nicotinamide regeneration all the required enzymes were functionally expressed, with FDH offering a specific activity of approximately 0.2 and 0.6 U mg^−1^ for PtDH. Concerning GDH, values ranged from 1.7 to 11.9 U mg^−1^ depending on the expression conditions used. However, GDH turned out to be the most active enzyme in terms of cofactor regeneration.

### Influence and toxicity of used substrates on the viability of *E. coli* W3110

**Fig. 3:**
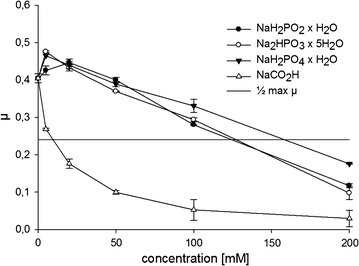
Toxicity study. Growth of *E. coli* W3110 in SGA medium supplemented with varying concentrations of sodium hypophosphite monohydrate (*black circle*), sodium phosphite dibasic pentahydrate (*white circle*), sodium phosphate monobasic monohydrate (*Black inverted triangle*) and sodium formate (*white triangle*). The maximum growth rate (μ) is plotted against the concentration of substrates and products

Substrates used in single-cell biotransformations as well as the corresponding obtained products might have toxic effects on the living host cell and thus limit the efficiency and turnover of the biocatalyst. The potential toxicity of several α-keto acids which are common substrates for transaminases AvtA and IlvE has already been assessed in previous work [49]. Since in the present study the single-cell catalyzed transamination reaction has been further investigated with an enzyme-coupled approach for cofactor regeneration, some more substrates had to be tested. For instance, PtDH from *Pseudomonas stuzeri* accepts phosphite or hypophosphite as substrate for the regeneration of NADH, whereas FDH from *Komagataella pastoris* requires formate. Therefore, sodium hypophosphite monohydrate, sodium phosphite dibasic pentahydrate, sodium phosphate monobasic monohydrate and sodium formate were added in varying concentrations to W3110 SGA minimal medium cultures at inoculation and growth was followed by Biolector^®^ cultivation system. Both phosphite substrates as well as phosphates had low inhibitory effects on *E. coli* W3110 cells in a similar range (Fig. 3). Even more than a half of maximum growth rate (µ) was observed at a concentration of 100 mM phosphite, hypophosphite or phosphate, respectively. In contrast, toxic effects based on addition of formate were more significant as the cell growth was reduced drastically by the presence of only 20 mM formate. Hence, the application of PtDH together with phosphite might be a promising way for regenerating consumed cofactors in single-cell catalyzed amination reactions.

### Fermentative amination of α-ketoacids

The successful assembly and co-expression of either transaminase AvtA or IlvE of module I with AlaDH or GluDH, respectively from module II gave rise to single-cell biocatalysts for one-pot transformations of α-ketoacids to α-amino acids in a fermentative fashion. In the first part of the study, cofactor regeneration was performed via the cell metabolism-coupled approach, using glucose as carbon source. As a second strategy, the enzyme-coupled strategy for cofactor regeneration was investigated and compared with previous results.

#### Fermentative amination of α-ketoacids with metabolic NADH regeneration

**Fig. 4:**
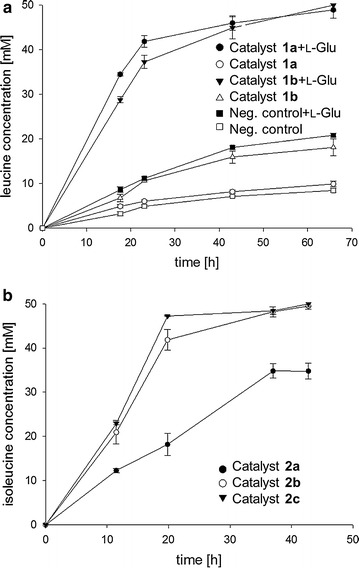
Fermentative amination of α-ketoacids with metabolic NADH regeneration. Fermentation of KIC to l-leucine **a** with catalyst 1a, 1b (*Ec*-GluDH carrying pTrc99A-*ilvE*_*Ec*_ or pTrc99A-*ilvE*_*Sm*_) and KMN to l-isoleucine **b** with catalyst 2a, 2b, 2c (*Ec*-AlaDH carrying pTrc99A-*avtA*, pTrc99A-*avta*-*ald* or pTrc99A-*ald*-*avtA*). SGA medium was inoculated aerobically with recombinant strains for the reductive amination of 50 mM KIC or KMV to l-leucine and l-isoleucine, respectively. 100 mM glucose, 50 mM (NH_4_)_2_SO_4_ and 0 or 250 mM l-glutamate in case of catalyst 1a–b were added to shift the reaction equilibrium towards product side

The first biocatalyst to reductively aminate α-ketoacids combined an endogenous (catalyst 1a) or a heterologous l-glutamate dependent transaminase IlvE from *Streptococcus mutans* (catalyst 1b), respectively with NAD(P)H-dependent l-glutamate dehydrogenase (GluDH) from *B. subtilis*. Using l-glutamate as amine donor, IlvE transforms several 2-ketoacids to the corresponding amino acids, among them being the three branched-chain 2-keto-isocaproate (KIC), 2-keto-isovalerate (KIV) and 2-keto-3-methylvalerate (KMV), which are converted to l-leucine, l-valine and to l-isoleucine, respectively. Since preliminary studies (data not shown) revealed that the enzyme has its highest affinity for KIC, the biotransformation of KIC to l-leucine catalyzed by newly assembled *E. coli* single-cell catalysts **1a** (*Ec*-GluDH/pTrc99A-*ilvE*_*Ec*_) and **1b** (*Ec*-GluDH/pTrc99A-*ilvE*_*Sm*_) was investigated with and without the addition of amine donor (Fig. 4a). As a negative control *Ec*-GluDH strain transformed with empty pTrc99A vector was used. The cofactor NADH was regenerated by oxidative catabolism of the employed carbon source glucose. Fermentative biotransformations were performed with 50 mM KIC and l-leucine formation was monitored over a time period of 66 h. While catalyst **1a** showed only a low conversion of 16% after 43 h, catalyst **1b** with IlvE from *Streptococcus mutans* converted twice as much (32%) KIC to l-leucine within the same time. Remarkably, the negative control *Ec*-GluDH/pTrc99A without overexpressed transaminase showed 14% turnover after 43 h as well. However, addition of 250 mM l-glutamate improved the conversion of all strains overexpressing a transaminase to 90–92%, while 36% conversion was obtained for the control strain after 43 h. Therefore, the presence of additional amine donor seems to play a key role under present reaction conditions, which might be explained by a too low expression level of GluDH and thus an insufficient regeneration of consumed l-glutamate.

The second single-cell biocatalyst for the fermentative production of amino acids relied on endogenous l-alanine dependent transaminase C (AvtA) co-expressed with NADH-dependent l-alanine dehydrogenase (AlaDH) from *B. subtilis*. These enzymes have already been investigated successfully for the single-cell catalyzed reductive amination of KMV to l-isoleucine using a two-plasmid based approach before [49]. In the present work, however, the concept was extended to a more flexible set-up with the *ald* gene being integrated into the host genome. Furthermore, the contribution of another *ald* gene copy being plasmid-encoded together with *avtA* as well as the effect of its cloning position was studied. It was assumed that the expression level of AlaDH played an important role in the turnover of the substrate. Biotransformations were again performed with 50 mM KMV in a fermentative way making use of the bacterial glucose catabolism for NADH recycling and the formation of l-isoleucine was monitored over 43 h (Fig. 4b). Employing catalyst **2a** (*Ec*-AlaDH/pTrc99A-*avtA*) for the fermentation, which offers only one genome-integrated copy of *ald*, moderate 68% of KMV were converted to l-isoleucine. However, when additional plasmid-born AlaDH was provided, conversion increased to 84% within 20 h using catalyst **2b** (*Ec*-AlaDH/pTrc99A-*avtA*-*ald*) and even 96% of product were formed with the help of catalyst **2c** (*Ec*-AlaDH/pTrc99A-*ald*-*avtA*) where a different gene order has been used. This strongly indicated that high expression of *ald* gene promoted the turnover of KMV to l-isoleucine, which coincided with the results obtained for catalyst **1**. The negative control *Ec*-AlaDH/pTrc99A did not produce any l-isoleucine. Due to the higher efficiency of the latter enzyme combination for reductive amination of α-ketoacids to α-amino acids, the single-cell catalyst composed of AvtA and AlaDH was chosen to be further investigated with the enzyme-coupled approach for cofactor regeneration based on GDH, FDH or PtDH.

#### Fermentative reductive amination of α-ketoacids with non-metabolic NADH regeneration

The tailor-made single-cell catalyst for reductive amination of KMV to l-isoleucine composed of AvtA and AlaDH was studied in more detail, using an enzyme-coupled approach for NADH cofactor regeneration instead of cellular oxidation of glucose. The gene for the required enzyme catalyzing the reoxidation of NAD^+^ to NADH was provided by module III and expressed using plasmid pBAD28∆*bla*, thus, it could be chosen independently from the other two enzymes. Three different strategies for cofactor regeneration were investigated: oxidation of formate to carbon dioxide by NAD^+^-dependent formate dehydrogenase (FDH), oxidation of glucose to β-d-glucono-1,5-lactone by glucose dehydrogenase (GDH) from *Bacillus megaterium* and oxidation of phosphite to phosphate by NAD^+^-dependent phosphite dehydrogenase (PtDH) from *Pseudomonas stutzeri*, respectively.

**Fig. 5:**
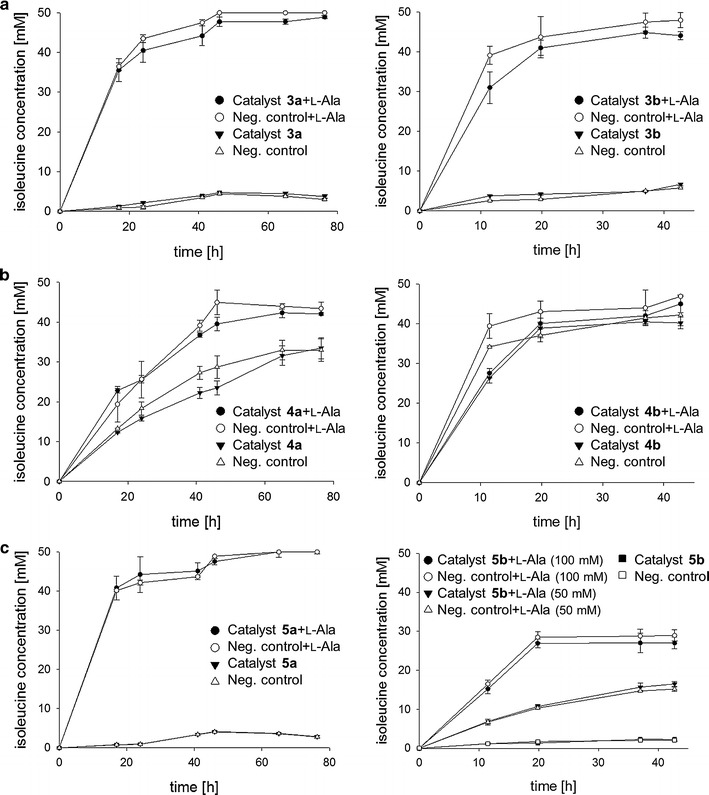
Fermentative amination of α-ketoacids with enzyme-coupled NADH regeneration. Fermentative reductive amination of KMV to l-isoleucine with *Ec*-AlaDH carrying pTrc99A-*avtA* (catalyst **3a**, **4a**, **5a**) or pTrc99A-*ald*-*avtA* (catalyst **3b**, **4b**, **5b**) and using either FDH (**a**), GDH (**b**) or PtDH (**c**) for cofactor regeneration. Strains were inoculated in SGA medium with OD_600_ 4 and 100 mM co-substrate, 50 mM (NH_4_)_2_SO_4_ and 0–250 mM l-alanine were added to push the reaction equilibrium towards product side

The reductive aminations were performed in a fermentative fashion (Fig. 5, Table 2), employing 50 mM KMV and 100 mM co-substrate. On the one hand, the three-enzyme-system was studied with the *ald* gene being localized in the genome and thus present as a single copy (catalysts **3a**, **4a** and **5a**). In that case, the addition of high amounts of l-alanine (250 mM) was necessary. On the other hand, experiments were performed with another *ald* gene copy (catalysts **3b**, **4b** and **5b**) introduced via plasmid pTrc99A-*avtA*. As already observed before, the *ald* gene being positioned right behind the P*trc* promoter (pTrc99A-*ald*-avtA) led to enhanced expression and activity, which is why this gene order was again used for additional AlaDH supply. In this case, the l-alanine concentration was reduced to 50–100 mM, as AlaDH expression and hence present amine donor amount was expected to be sufficient. For control reactions the respective strain was transformed with empty pBAD28∆*bla* lacking the gene for cofactor regeneration, resulting in a fermentative amination which makes use of metabolic NADH regeneration and hence corresponds to catalysts **2a**, **2b** and **2c**, respectively. Coupling the l-alanine dependent reductive amination catalyzed by AvtA with an enzyme catalyzed NADH regeneration system did not improve the overall conversion of KMV to l-isoleucine in any case compared with the cellular regeneration approach (control strains). No significant turnover at all was achieved neither with the FDH-coupled catalyst **3a** (8% after 41 h), nor with the PtDH-coupled **5a** (7% after 41 h). However, 44% conversion was achieved in the same reaction time with catalyst **4a**, using GDH as recycling enzyme. This result coincided with the higher enzyme activities measured for GDH in contrast to the other recycling enzymes. Interestingly, comparable or even higher conversions were obtained in all three cases with corresponding control strains, indicating that the expression level of regeneration enzymes is not sufficient to outperform the cell-driven NADH recycling based on glucose (Table 2).

**Table 2:** Obtained results for the reductive amination of KMV with and without addition of amine donor l-alanine

Catalyst	Conversion [%] without l-alanine		Conversion [%] with l-alanine	
Cofactor regeneration	Enzyme-coupled	Metabolic	Enzyme-coupled	Metabolic
**3a** (*Ec*-AlaDH/pTrc99a-*avtA*/pBADΔ*bla*-*fdh*)	8	8	88^a^	94^a^
**3b** (*Ec*-AlaDH/pTrc99a-*ald*-*avtA/*pBADΔ*bla*-*fdh*)	13	12	88^b^	96^b^
**4a** (*Ec*-AlaDH/pTrc99a-*avtA*/pBADΔ*bla*-*gdh*)	44	54	73^a^	78^a^
**4b** (*Ec*-AlaDH/pTrc99a-*ald*-*avtA/*pBADΔ*bla*-*gdh*)	80	84	90^b^	94^b^
**5a** (*Ec*-AlaDH/pTrc99a-*avtA*/pBADΔ*bla*-*ptdh*)	7	7	90^a^	87^a^
**5b** (*Ec*-AlaDH/pTrc99a-*ald*-*avtA/*pBADΔ*bla*-*ptdh*)	5	6	54^c^	58^c^

Conversion obtained for the enzyme-coupled system for cofactor regeneration was compared with the metabolic one

^a^Addition of 250 mM l-alanine

^b^Addition of 50 mM l-alanine

^c^Addition of 100 mM l-alanine

Moreover, again the genome-based *ald* expression was expected to be a limiting parameter for regeneration of consumed amine donor and thus for an efficient reductive amination. Indeed, the addition of 250 mM l-alanine increased overall turnover significantly both for all single-cell catalysts as well as for control strains, leading to 88–90% of product formation for catalyst **3a** and **5a** and 73% for **4a**. An additional cloning of *ald* on the plasmid pTrc99A-*ald*-*avtA* did not increase the catalytic efficiency of the *E. coli* cells considerably. While a turnover of 13% and comparable 11.5% were achieved with catalyst **3b** and its control strain after 42 h, catalyst **5b** led even to reduced product formation (5%). The only exception turned out to be catalyst **4b** making use of a GDH, where an additional *ald* gene copy seemed to be beneficial and increased turnover of KMV from 44 to 80%. Nevertheless, comparable turnover was obtained with the control strain too. Once more, a remarkable enhancement was possible when additional amine donor (50 mM) was present in the fermentation broth, leading to 88% product formation with catalyst **3b**. Similarly, turnover of catalyst **4b** could be slightly increased to 90%. However, still moderate conversions were achieved with catalyst **5b**, even when 50 or 100 mM l-alanine were added, resulting in 17 and 54% conversion, respectively.

### “In vitro-type” single-cell catalyzed amination of prochiral ketones

**Fig. 6:**
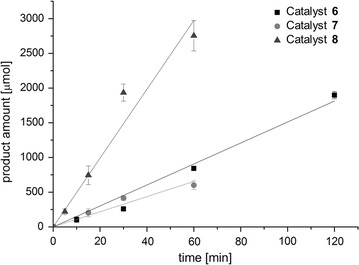
Activities of singe-cell catalysts for “in vitro-type” amination of ketones. Initial rates of single-cell catalysts **6** (*Ec*-AlaDH/pTrc99A-*ta*_*Cv*_/pBAD28Δ*bla*-*fdh*), **7** (*Ec*-AlaDH/pTrc99A-*ta*_*Cv*_/pBAD28Δ*bla*-*gdh*) and **8** (*Ec*-AlaDH/pTrc99A-*ta*_*Cv*_/pBAD28Δ*bla*-*ptdh*) in the reductive amination of 4-phenyl-2-butanone (25 mM), coupling Ta_Cv_ with AlaDH and either FDH, GDH or PtDH for cofactor regeneration. Typical time curves are shown for the production of 4-phenyl-2-butylamine over a time period of 60 or 120 min, respectively using 50 mg resting cells per mL

**Fig. 7:**
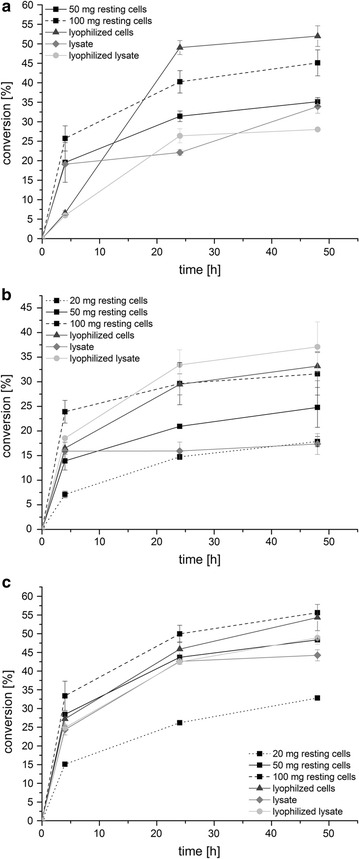
"In vitro-type" amination of a prochiral ketone. Investigation of catalyst **6** (**a**), catalyst **7** (**b**) and catalyst **8** (**c**) in the reductive amination of 4-phenyl-2-butanone over 48 h. The biocatalyst was either applied as resting cells (*black square*), as lyophilized cells (*black triangle*), as cell-free extract (*grey diamond*) or as lyophilized cell-free extract (*grey circle*), respectively and the reaction was monitored over 48 h

Besides enabling the single-cell catalyzed reductive amination of α-ketoacids in a one-pot fermentation, the applicability of the modular plug-and-play concept was furthermore demonstrated for the synthesis of optically pure amines. For this purpose, a transaminase from *Chromobacterium violaceum* (Ta_Cv_) from module I offering a broader substrate spectrum was coupled with AlaDH from module II for regenerating the consumed amine donor l-alanine as well as with each of the three available enzymes from module III for NADH cofactor recycling, resulting in three single-cell catalysts **6**–**8** (see Table 1). The asymmetric reductive amination of 4-phenyl-2-butanone was chosen as model reaction and the overall performance of each single-cell biocatalyst was compared by determining initial rates (Fig. 6) for the mentioned transformation. With respect to catalyst **6**, co-expressing Ta_Cv_ together with AlaDH and FDH, an apparent overall activity of 0.30 ± 0.02 U g^−1^ was obtained, whereas catalyst **7** involving a GDH for cofactor regeneration showed an apparent activity of 0.22 ± 0.02 U g^−1^. The best result, however, was obtained for catalyst **8**, combining the transamination machinery with a PtDH and reaching thereby an apparent activity of 0.99 ± 0.07 U g^−1^. Then the reactions were monitored over a time period of 48 h, employing and comparing different catalyst preparations (Fig. 7). Besides the use of resting cells, the biocatalyst was applied in the form of lyophilized single-cells, cell-free extract and lyophilized cell-free extract. For sake of better comparability the amount of used lyophilized cells, lysate and lyophilized lysate always correlated to 50 mg of wet cells. Employing resting cells of catalyst **6** in the model reaction (Fig. 7a) led to formation of 35% of the desired amine, which was slightly increased to 45% with a twofold catalyst loading (100 mg mL^−1^). Similar results were obtained with catalyst **8**, yielding already 33% of amine with half the catalyst amount (20 mg mL^−1^), which could be increased to 48–56% with one- to twofold of catalyst loading, respectively (Fig. 7c). Less efficient in the reductive amination of 4-phenyl-2-butanone seemed to be catalyst **7**, converting only 25–32% of the ketone depending on the amount of used resting cells (50–100 mg mL^−1^). However, with catalyst **7** the occurrence of limitations during the reaction time became rather obvious (Fig. 7b), as within the first 4 h the reaction rate was accelerated proportional to the amount of employed catalyst, but with proceeding reaction this correlation decreased significantly and almost no amine was formed anymore after 24 h. With respect to GDH-catalyzed cofactor regeneration, especially local acidification within the cell caused by the co-product gluconic acid might be problematic, presumably leading to enzyme inactivation. In general, applying the biocatalyst as lyophilized single-cells (corresponding to 50 mg of wet cells) significantly improved conversion levels in all cases, giving 52, 33 and 54% of amine with catalyst **6**, catalyst **7** and catalyst **8**, respectively. Most likely, the cell membrane was permeabilized due to lyophilization in a way that led to better access of reaction components. Surprisingly, performing the biotransformation either with cell-free or lyophilized cell-free catalyst preparations, respectively did not show further beneficial effect on the outcome of the reaction. This was at least the case for catalyst **6** and **8**. In contrast, the highest product formation (37%) with catalyst **7** was achieved when employed as lyophilized cell-free extract. The inability of *E. coli* cells to import glucose without permeabilization of the cell membrane is a major drawback of GDH-catalyzed cofactor regeneration [50], which is why the complete removal of the cell wall might be improving the overall catalyst performance. In order to further increase the amine production by the recombinant *E. coli* single-cell catalyst, the effect of a higher catalyst loading using catalyst **6** in form of lyophilized cells was investigated. With a tenfold amount of catalyst, conversion to the amine could be increased by a factor of 1.5 to maximum 77% within 48 h. This indicated once more the occurrence of limitations over time such as enzyme inactivation or inhibition. As already observed during one-pot fermentations of α-ketoacids before, also a too low activity level of AlaDH might lead to an inefficient reaction equilibrium and thus, to not quantitative conversions. Nevertheless, the concept of assembling the reaction cascade for reductive amination of prochiral ketones in vivo within one *E. coli* cell has been successfully proven, affording the (*S*)-amine in high yields with excellent to perfect optical purity (92–99%).

## Discussion

“Tailor-made” single-cell biocatalysts co-expressing multiple enzymes enable efficient in vivo reaction cascades [28, 32]. Consequently, the design and application of such microbial cell factories has attracted attention in recent years, aiming for the production of a broad range of valuable chiral compounds. Since synthetic pathways commonly may involve cofactor-dependent redox reactions [51], various studies focused on the incorporation of reaction pathways in a host cell whereby the sequence itself enables a suitable cofactor regeneration resulting in redox self-sufficient single-cell catalysts [42, 52]. Alternatively, recycling of cofactors can be achieved by making use of the host’s inherent metabolic pathways, e.g. the catabolism of carbon sources like glucose [53]. In this study, we established a modular platform to construct *E. coli* single-cell biocatalysts tailored for the in vivo amination of ketoacids and prochiral ketones, exploiting both cell metabolism as well as additional enzyme-mediated strategies for cofactor regeneration. The fermentative transformation of KMV to l-isoleucine showed that the use of an enzyme-coupled approach for NADH regeneration led to similar conversion as obtained with the metabolism-coupled one. This strongly suggests that the expression level of regenerating enzymes FDH, GDH and PtDH, respectively, was insufficient, lowering the overall catalytic performance. As a matter of fact, impaired enzyme production is a well-known obstacle of whole-cell biocatalysis caused by an increased metabolic burden during cell growth due to the co-expression of multiple proteins within one cell [31]. In case of the amino acid dehydrogenases GluDH and AlaDH a low expression level became apparent too, since extra addition of amine donors l-glutamate and l-alanine, respectively, to the fermentation broth significantly improved the transformation of both KIC and KMV to the corresponding α-amino acids. The role of l-alanine in such in vivo amination cascades has already been investigated before concerning the redox self-sufficient amination of alcohols [43]. Since l-alanine is not only required as amine donor for the desired reaction but also as energy source for the cell to maintain viability and the protein biosynthesis machinery under stress conditions, its addition in certain amounts is to be recommended, or even necessary. However, it was possible to improve the amine donor regeneration system by including a second *ald* gene copy additionally to the one being genome-integrated. This change on the genetic level led to higher amounts of expressed AlaDH and consequently to increased product formation, confirming once more the assumption of reduced catalyst performance due to impaired enzyme synthesis. However, not only the additional plasmid-born *ald* gene but also its gene order contributed to enhanced protein expression. This emphasized the crucial role of a thoroughly reasoned co-expression strategy in order to fine-tune expression levels and to achieve a functional microbial cell as catalytic unit. A vast synthetic biology toolbox is available for that purpose, offering different promoter systems, plasmid types and strategies to compose the ideal co-expression cassette [28, 54, 55].

In this study, a recombinant two-plasmid-based gene expression was combined with a genome-integrated one, ideally enabling control and induction of protein production independent from the host’s regulatory network. Furthermore, this modular system allowed for easy substitution of the individual enzymes according to the desired application of the single-cell catalyst. Exchanging for example the l-alanine–valine aminotransferase with the (*S*)-selective transaminase from *Chromobacterium violaceum* facilitated a broader substrate range and hence, a widened applicability of the approach. Thus, additionally to the amination of α-keto acids also the transformation of a prochiral ketone into an optically active amine was achieved. The amination of 4-phenyl-2-butanone was performed in a metabolism-independent fashion, making use of enzyme-coupled regeneration of redox cofactors. In such “in vitro-type” reactions decoupled from the host’s metabolism parameters like activity, stability and concentration of enzymes co-expressed within the single-cell biocatalyst play a particularly important role [54]. Accordingly, limitations were observed during the amination of 4-phenyl-2-butanone, which might be attributed again to an insufficient and imbalanced enzyme expression. Next to a generally low enzymatic activity of AlaDH and NADH recycling enzymes also a loss thereof over time caused by inactivation or inhibition might be an issue. The adaption of the cellular metabolism of resting cells to the non-growth status results in a restricted self-regeneration and stress-handling capacity, leading to a decrease of active enzyme amounts and as a consequence to an intracellular NADH shortage [56]. This might have been the reason why it was not possible to perform the “in vitro-type” reactions satisfyingly without the external addition of cofactors. Conversions were much higher when NAD^+^ was present in the reaction mixture. According to literature the NAD^+^ level of *E. coli* lies in the range of 5.3 nmol mg^−1^ dry weight under standard growth conditions [57]. Thus, the application of whole-cell biocatalysts without additional coenzyme may be possible, but it has been reported before that by adding coenzymes the efficiency of the reaction is significantly enhanced [58]. Another crucial parameter in terms of non-fermentative transformations turned out to be the permeabilization of the cell wall. Mass transfer limitations were overcome by lyophilization or even complete removal of cell wall via disruption and thus, higher conversions were obtained for the amination of 4-phenyl-2-butanone.

## Conclusions

The developed concept of a plug-and-play *E. coli* single-cell biocatalyst provides a library of enzymes for each of the different required reactions/modules of a reductive amination and suitable *E. coli* hosts, which can be combined depending on the desired target reaction. Consequently, it represents a quite promising and cost-efficient alternative to the combination of isolated enzymes. Especially, from an industrial point of view the described approach offers major advantages like the decrease of required fermentations and the reduction of costly and laborious isolation and purification steps. The flexible approach can be easily extended to other transaminases, allowing for a quick identification of the most suitable single-cell catalyst tailored for a specific substrate or a desired stereoselectivity. Depending on the required co-substrate the appropriate amino acid dehydrogenase as well as NADH recycling system can be chosen. A further improvement of the modular platform would be the addition of an alanine racemase, enabling the in situ racemization of l-alanine to d-alanine and thus the efficient use of (*R*)-selective ω-TAs. In summary, the presented approach offers a platform to construct a broad range of single-cell catalysts in a flexible plug-and-play fashion for reductive amination. In comparison with the in vitro transamination system using individual biocatalysts from separate preparations, the single-cell approach enables easy production of the required enzymes and cost-effective biotransformations.

## Methods

### Construction of bacterial strains and plasmids

Bacterial strains and plasmids used in this study are summarized in Table 3. Plasmid pRed/ET (tet^R^) was obtained from the “Quick and Easy *E. coli* Gene Deletion Kit”, Gene Bridges (Heidelberg, Germany) and plasmid pQE30 was received from Qiagen (Hilden, Germany). Plasmid isolation was performed with the QIAprep spin miniprep kit (Qiagen, Hilden, Germany). Standard DNA work like polymerase chain reaction (PCR), restriction and ligation were performed as described previously [59]. The oligonucleotides used in this study were obtained from Metabion GmbH (Martinsried, Germany) or Eurofins Genomics and are listed in Additional file 1: Table S1. For transformation CaCl_2_-competent *E. coli* cells [60] were heat-shocked for uptake of respective DNA. All cloned DNA fragments were shown to be correct by sequencing performed by LGC Genomics GmbH (Berlin, Germany).

**Table 3:** List of bacterial strains and plasmids used in this work

	Relevant characteristics	Source/references
Strains		
*E. coli* TOP10	F^−^ *mcrA* Δ(*mrr*-*hsd*RMS-*mcr*BC) Φ80*lac*ZΔM15 Δ *lac*X74 *rec*A1 *ara*D139 Δ(*araleu*)7697 *gal*U *gal*K *rps*L (StrR) *end*A1 *nup*G	Invitrogen
*E. coli* W3110	F^−^ λ^−^ INV(*rrn*D-*rrn*E)1 *rph*-1	[61]
*E. coli* AlaDH	W3110*ΔaraBAD*::P*trc*-*ald, gene for alanine dehydrogenase (ald)* from *Bacillus subtilis* D196A/L197R integrated into *ara* locus in the choromosome and expressed from P*trc* promoter	This study
*E. coli* GluDH	W3110*ΔaraBAD*::P*trc*-*rocG, gene for glutamate dehydrogenase (rocG)* from *Bacillus subtilis* D196A/L197R integrated into *ara* locus in the choromosome and expressed from P*trc* promoter	This study
Plasmids		
pTrc99A	P*trc*, pBR322ori, *rrnB* T1, *rrnB* T2, *lac* ^*Iq*^, *bla*, template for P*trc* Promoter	[47]
pTrc99A-*avtA*	pTrc99A carrying l-alanine–valine aminotransferase (*avtA*) from *E. coli* MG1655	[49]
pTrc99A-*avtA*-*ald*	pTrc99A carrying *avtA* from *E. coli* MG1655, *ald* from *Bacillus subtilis*	[49]
pTrc99A-*ald*-*avtA*	pTrc99A carrying *ald* from *Bacillus subtilis*, *avtA* from *E. coli* MG1655	This study
pTrc99A-*ilvE*_*Ec*_	pTrc99A carrying branched-chain amino-acid aminotransferase (*ilvE*) from *E. coli* MG1655	This study
pTrc99A-*ilvE*_*Sm*_	pTrc99A carrying *ilvE* from *Streptococcus mutans*	This study
pTrc99A-ta_*Cv*_	pTrc99A carrying (*S*)-selective ω-transaminase from *Chromobacterium violaceum*	This study
pBAD28	P*ara*, p15Aori, *rrnB* T1, *rrnB* T2, *bla,* Cam^R^	[48]
pBAD28*Δbla*	pBAD28 with deletion of β-lactamase gene	This study
pBAD28*Δbla*-*fdh*	pBAD28*Δbla* carrying formate dehydrogenase *(fdh*) from *Komagataella pastoris GS115*	This study
pBAD28*Δbla*-*gdh*	pBAD28*Δbla* carrying glucose dehydrogenase (*gdh*) from *Bacillus megaterium*	This study
pBAD28*Δbla*-*ptdh*	pBAD28*Δbla* carrying *ptdh* from *Pseudomonas stuzeri*	This study
pQE30	phage P*T5* promoter, Col E1, 6xHis, *bla*, template for P*T5*	Qiagen
pKD13	*oriRΥ, bla,* Km^R^, template for kanamycin cassette	[62]
pTrc99A-*kan*-P*trc*-*ald*	pTrc99A carrying FRT-flanked *kan* resistance gene of pKD13, P*trc* Promoter, *ald* from *Bacillus subtilis*	This study
pTrc99A-*kan*-P*trc*-*rocG*	pTrc99A carrying FRT-flanked *kan* resistance gene of pKD13, P*trc* Promoter, *rocG* from *Bacillus subtilis*	This study
pRed/ET	*Red* recombinase expression plasmid (ts), pSC101 based, Tc^R^	Gene bridges
pCP20	*RepA101*(ts), *bla*, λ-red Flp recombinase for removal of resistance cassette	[62]

#### Construction of expression plasmids

Plasmids were constructed with fragments generated by PCR (KOD Hot Start Polymerase kit, Novagen, Darmstadt, Germany) using *Bacillus subtilis* as template for *ald* and *rocG* genes, *E. coli* MG1655 and *Streptococcus mutans* for *ilvE* genes, *Chromobacterium violaceum* for *ta* gene, *Komagataella pastoris* GS115 for *fdh* gene, *Bacillus megaterium* for *gdh* gene, *Pseudomonas stuzeri* for *ptdh* gene and *E. coli* W3110 for genes *phnC, phnD, phnE*. In this study two different cloning strategies based on the IPTG-inducible *E. coli* expression vector pTrc99A were used. In the first strategy the particular gene was inserted into the vector by using cut sites. In order to construct pTrc99A-*ald*-*avtA*, the oligonucleotides *ald*_*Bs*__RBS_fw/*ald*_*Bs*__rv were used and the PCR product was ligated into the vector pTrc99A-*avtA* via *Eco*RI restriction site. The construction of pTrc99A-*ilvE*_*Ec*_ and pTrc99A-*ilvE*_*Sm*_ was performed similarly with the primers *ilvE*_*Ec*__RBS_fw/*ilvE*_*Ec*__rv, *ilvE*_*Sm*__RBS_fw/*ilvE*_*Sm*__rv and *Eco*RI restriction site. For pTrc99A-*ta*_*Cv*_ the PCR-amplified gene product using *ta*_*Cv*__fw/*ta*_*Cv*__rv was cut with *Eco*RI and *Sal*I and then ligated into pTrc99A digested with the same restriction enzymes. In the second strategy the Gibson assembly method [63] was performed as a second cloning strategy to construct pTrc99A-*ald*-*avtA*-*ptdh.* For this purpose the genes *ald* of *Bacillus subtilis*, *avtA* of *E. coli* MG1655 and *ptdh* of *Pseudomonas stuzeri* were amplified with the oligonucleotides GA_*ald*_RBS_*Eco*RI_fw/GA_*ald*_rv, GA_*avtA*_RBS_fw/GA_*avtA*_rv and GA_*ptdh*_RBS_fw/GA_*ptdh*_*Xba*I_rv, respectively and assembled with *Eco*RI/*Xba*I restricted pTrc99A using Gibson assembly method [63].

For expression of the genes encoding NAD(P)H-regenerating enyzmes the arabinose-inducible pBAD28 plasmid was used. For deletion of the *bla* gene, the plasmid was digested with the restriction enzymes *Alw44*I and *Nsb*I, treated with Klenow fragment and ligated. Subsequently, the obtained plasmid pBAD28∆*bla* was used for generating plasmids pBAD28∆*bla*-*fdh* (*fdh*_RBS_fw/*fdh*_rv), pBAD28∆*bla*-*gdh* (*gdh*_RBS_fw/*gdh*_rv) and pBAD28∆*bla*-*ptdh* (*ptdh*_RBS_fw/*ptdh*_rv). Before ligation, the pBAD28∆*bla* vector as well as the PCR-derived gene products were treated with *Sac*I and *Xba*I enzymes.

#### Construction of bacterial strains harbouring amino acid dehydrogenase genes

The open-reading coding region of the genes *araBAD* from *E. coli* W3110 genome were replaced with the gene *ald* or *rocG* under control of IPTG-inducible P*trc* promoter and a kanamycin cassette flanked by FLP recognition target sites by using modified one-step method for inactivation of genes [62]. For the construction of recombination plasmids, again the Gibson assembly method was applied. Linear DNA-fragments comprising FLP-*kan*-FLP cassette and P*trc* promoter as well as *ald* or *rocG* gene, respectively were obtained by PCR using primers GA_*kan*_*Eco*RI_fw/GA_*kan*_rv, GA_trc_fw/GA_trc_rv, GA_*ald*_RBS_fw/GA_*ald*_*Eco*RI_rv, GA_*rocG*_RBS_fw/GA_*rocG*_*Eco*RI_rv. For this purpose pTrc99A or pQE30 plasmid were used and genomic DNA from *B. subtilis* and pKD13, respectively as template. The linear pTrc99A vector (cut with *Eco*RI) and overlapping DNA fragments were assembled to pTrc99A-*kan*-P*trc*-*ald* and pTrc99A-*kan*-P*trc*-*rocG* plasmids. All gene cassettes were amplified by PCR using primers with homologous arms consisting of 50 nucleotides upstream (HS_*araCB*_fw) and downstream (HS_*araD*_rv) of the *araBAD* genes. The corresponding 3-kbp PCR products were purified, treated with *Dpn*I, and then transformed by electroporation into *E. coli* W3110(Red/ET) using “Quick and Easy *E. coli* Gene Deletion Kit” (Gene Bridges, Heidelberg) according to the manual provided by the supplier. Cells with homologous recombination were selected on an agar plate containing kanamycin and screened by colony PCR with *araC*_fw/Kt_rv primers. The antibiotic marker was removed by using a helper plasmid pCP20 encoding FLP-recombinase. The Km^R^ mutants were transformed with temperature-sensitive plasmid pCP20 and Amp^R^ transformants were selected at 30 °C. The elimination of antibiotic marker was verified by PCR (*araC*_fw/*polB*_rv). The helper plasmid was removed by strike out on non-selective plates at 43 °C. Newly obtained mutation strains were designated *Ec*-AlaDH *(E. coli* W3110Δ*araBAD*::P*trc*-*ald)* and *Ec*-GluDH *(E. coli* W3110Δ*araBAD*::P*trc*-*rocG).* For fermentative production experiments strain *Ec*-AlaDH was transformed with plasmids pTrc99A-*avtA*, pTrc99A-*avtA*-*ald* or pTrc99A-*ald*-*avtA* and pBAD28Δ*bla*, *pBAD28*Δ*bla*-*fdh*, *pBAD28*Δ*bla*-*gdh* or *pBAD28*Δ*bla*-*ptdh*, respectively. Alternatively, they were transformed with pTrc99A-*ald*-*avtA*-*ptdh* and pBAD28Δ*bla*-*phnCDE*. Strain *Ec*-GluDH was transformed with plasmids pTrc99A-*ilvE*_*Ec*_ or pTrc99A-*ilvE*_*Sm*_. For preparing the single-cell catalysts used for “*in vitro*-type” reactions, strain *Ec*-AlaDH was transformed with plasmids pTrc99A-*ta*_*Cv*_ and *pBAD28*Δ*bla*-*fdh*, *pBAD28*Δ*bla*-*gdh* or *pBAD28*Δ*bla*-*ptdh*, respectively.

### Media and cultivation conditions

*Escherichia coli* W3110 cells were grown in lysogeny broth (LB) complex medium (10 g L^−1^ of tryptone, 5 g L^−1^ of yeast extract, 10 g L^−1^ of sodium chloride) at 37 °C in baffled Erlenmeyer flasks (60–1000 mL) on a rotary shaker at 120 rpm. Ampicillin (100 mg L^−1^), kanamycin (50 mg L^−1^) and/or chloramphenicol (25 mg L^−1^) were added when appropriate. The growth of *E. coli* was monitored by measuring the optical density at 600 nm (OD_600_). Protein expression was induced at an OD_600_ of approximately 0.5–0.7 by the addition of isopropyl-β-d-thiogalactopyranoside (IPTG, 0.25–1 mM) and/or l-arabinose (0.02–0.3 vol%). Afterwards shaking was continued over night at 120 rpm and 20, 25, 30 or 37 °C, respectively. The cells were then harvested by centrifugation (2600–10,000×*g* for 10–30 min at 4 °C) and the resulting cell pellet was either applied directly in single-cell biotransformations or stored at −20 °C until further use. By lyophilizing the single-cell biocatalyst, its storage at 4 °C was possible for several weeks without any significant loss of enzyme activity. For this purpose the cell pellet was resuspended in a minimum amount of sodium phosphate buffer (NaP_i_, 50 mM, pH 8, 0.5 mM PLP), frozen in liquid nitrogen and lyophilized over night.

### Preparation of cell-free extracts

For preparation of cell-free extract the harvested cells were resuspended in NaP_i_ buffer (50 mM, pH 8) yielding a 15 wt% cell solution. Cells were lysed by sonication treatment at 4 °C (15–60, 0.1 s sonicate, 0.4 s pause, 40% amplitude using a Branson Digital Sonifier^®^) and crude extract was centrifuged (13,000–16,000× for 10–30 min at 4 °C). The remaining cell debits were resuspended (15 vol%) in urea (6 M) for SDS-analysis. The supernatant was kept at 4 °C to be either applied directly in biotransformations or to be analyzed by activity assays and SDS-PAGE. Otherwise, the supernatant was frozen in liquid nitrogen and lyophilized overnight yielding in lyophilized cell-free extract.

### Fermentative production of l-isoleucine and l-leucine

For the production of l-isoleucine and l-leucine strains derived from *E. coli* W3110 were grown in LB medium supplemented with 1 mM IPTG and/or 0.3% l-arabinose at 37 °C and 200 rpm. Then the cells were washed twice with the medium salts (49 mM KH_2_PO_4_; 76 mM K_2_HPO_4_ for FDH and GDH or 125 mM H_3_O_3_P for PtDH), resuspended and cultivated with an OD_600_ of four in a chemically defined synthetic glycerol ammonium sulfate (SGA) medium as described previously [49]. As carbon source either 100 mM glucose for GDH, 100 mM sodium formate for FDH or 100 mM sodium hypophosphite for PtDH were used instead of glycerol. In the case of GDH the cells were cultured anaerobically for the production of l-isoleucine. When using PtDH, the phosphate components were replaced by phosphite components. For induction of expression 1 mM IPTG and/or 0.3% l-arabinose were added to the cultures. For production of l-isoleucine 50 mM MOPS, 100 mM (NH_4_)_2_SO_4_, 50 mM 2-keto-3-methylvalerate (KMV) and 0–250 mM l-alanine were added to the medium. For production of l-leucine 50 mM MOPS, 100 mM (NH_4_)_2_SO_4_, 50 mM 2-keto-isocaproate (KIC) and 0–250 mM l-glutamate were added to the medium. For quantification of extracellular amino acids, aliquots of the culture were taken, cells were removed by centrifugation at 13,000×*g* for 10 min, and the supernatants were frozen at −20 °C.

### Toxicity tests

For the study of substrate toxicity batch cultivations with *E. coli* W3110 were performed in the Biolector^®^ cultivation system (m2p Labs, Baesweiler) using 1 mL medium microtiter plates (Flower Plate^®^, m2p Labs, Baesweiler) at 1100 rpm at 37 °C. The cells were grown in SGA minimal medium with 1% Glucose and various concentrations (5, 20, 50, 100 and 200 mM) of sodium hypophosphite monohydrate (NaH_2_PO_2_ × H_2_O), sodium phosphite dibasic pentahydrate (Na_2_HPO_3_ × 5H_2_O), sodium phosphate monobasic monohydrate (NaH_2_PO_4_ × H_2_O) and sodium formate (NaCO_2_H). The experiments were carried out in triplicates.

### Activity assays

All assays were performed with cell-free extracts in triplicates and one unit of enzyme activity (U) was calculated as the amount of enzyme catalyzing the conversion of 1 µmol of substrate in 1 min. Protein concentration was determined by the Bio-Rad Protein Assay based on the method of Bradford [64] using bovine serum albumin as a reference standard.

#### Measurement of transaminase activity

The activities of AvtA and IlvE were measured as described before [65] using 10 mM keto acid (2-keto-3-methylvalerate (KMV) or 2-keto-isocaproate (KIC) and 50 mM amino donor (l-alanine for AvtA or l-glutamate for IlvE, respectively). The determination of Ta_Cv_ activity was performed via an indirect photometric assay based on the reductive amination of pyruvate to l-alanine using (*S*)-methylbenzylamine as amine donor. For this purpose a substrate solution (990 µL) containing PLP (0.1 mM), (*S*)-methylbenzylamine (10 mM) and sodium pyruvate (5 mM) in NaP_i_ buffer (50 mM, pH 8) was mixed with cell-free extract (10 µL) in a semi-micro UV-cuvette. Immediately, the increase of acetophenone formation (initial rate Δc/Δt) was measured over time at 290 nm.

#### Measurement of activities of amino acid dehydrogenases

The activities of AlaDH and GluDH were assayed based on the conversion of pyruvate to l-alanine with concomitant NADH consumption, which was monitored spectrophotometrically at 340 nm as described before [65].

#### Measurement of activities of NADH-recycling enzymes

The increase of NADH absorption at 340 nm was used for determining activities of NADH-recycling enzymes. FDH-activity was calculated based on the oxidation of formate with concomitant reduction of NAD^+^ to NADH. For measuring the activity of GDH β-d-glucose was converted to d-glucono-1,5-lactone and in order to assaying PtDH activity phosphite was oxidized to phosphate. In all cases cell-free extract (10 µL) was added to a substrate solution (970 µL) containing NAD^+^ (1 mM) and either ammonium formate (100 mM), d-glucose (100 mM) or sodium phosphite (100 mM) in NaP_i_ buffer (50 mM, pH 8) and the increase of NADH absorption at 340 nm over time (initial rate Δc/Δt) was measured immediately.

### Biotransformations

Biotransformations were performed in NaP_i_ buffer (50 mM, pH 8, 1 mM PLP) with l-alanine as amine donor and a substrate concentration of 25 mM. The particular co-substrate was applied according to the used cofactor-recycling enzyme. Together with the FDH catalyzed recycling system ammonium formate was used, while for the GDH system glucose and for the PtDH system sodium phosphite was required. For the GDH and the PtDH system additionally ammonium acetate was needed as nitrogen donor. The recombinant *E. coli* catalyst was employed either in form of resting cells (50 mg resuspended in 500 µL reaction buffer) or as cell-free extract (~350 µL, corresponding to 50 mg wet cells mixed with 150 µL reaction buffer) in an Eppendorf tube (1.5 mL). Alternatively, lyophilized single-cells (corresponding to 50 mg wet cells) or lyophilized cell-free extract (corresponding to 350 µL lysate) were rehydrated in reaction buffer for 15 min at 30 °C and 120 rpm prior to use. Then, reaction buffer (500 µL) containing l-alanine (250 mM, 5 eq), NAD^+^ (2 mM), the particular co-substrate (300 mM, 6 eq) and ammonium acetate (150 mM, 3 eq) in case of GDH and PtDH recycling system was added. The reaction was started by the addition of the substrate 4-phenyl-2-butanone (3.8 µL, 25 mM) and the mixture was shaken at 30 °C and 800 rpm for up to 48 h using an orbital shaker. After 4, 24 and 48 h, respectively 200 µL of each sample were withdrawn and the reaction was quenched with aqueous NaOH solution (20 µL, 10 M). After extraction with EtOAc (2 × 400 µL) the combined organic layers were dried over Na_2_SO_4_, and conversion as well as *enantiomeric excess* (*ee*) were analyzed by GC.

#### Determination of initial rates of single-cell catalysts

The activity of the recombinant *E. coli* resting cells for the reductive amination of 4-phenyl-2-butanone to the corresponding amine was assayed over 1–2 h. For this purpose the biotransformation was performed according to the procedure described above. The reaction was stopped after 5, 10, 20, 40, 60 and 120 min, respectively by adding aqueous NaOH solution (20 µL, 10 M) and extracted with EtOAc (2 × 400 µL). The combined organic layers were dried over Na_2_SO_4_ and conversions were analyzed by GC. The enzyme activity (U) was defined as the amount of enzyme that catalyzes the conversion of 1 µmol of substrate per minute.

### Analytical methods

#### Determination of conversion

Quantification of amino acid content in the fermentation reactions was performed using a high-pressure liquid chromatography system (HPLC, 1200 series, Agilent Techologies Deutschland GmbH, Böblingen, Germany) and an automatic precolumn derivatization with *ortho*-phthaldialdehyde. The amino acids were separated on a reversed phase column as described previously [66]. The conversion of 4-phenyl-2-butanone to 4-phenyl-2-butylamine was determined by GC-analysis (see chromatograms in Additional file 1: Figures S1, S2) on an Agilent 7890 A GC system equipped with a flame ionization detector (FID) using H_2_ as carrier gas. Ketone and amine were separated on an achiral stationary phase using a 14% cyanopropylphenyl phase capillary column (J&W Scientific DB-1701; 30 m × 250 µm × 0.25 µm) with an injection and detection temperature of 250 °C (temperature program: 120 °C, 10 °C min^−1^ to 180 °C, 60 °C min^−1^ to 280 °C, hold 2 min).

#### Determination of enantiomeric excess

For the determination of the enantiomeric excess (*ee*) the regarding samples were derivatized by incubating them with pyridine (2 eq) and acetic anhydride (5 eq) for 2 h at 40 °C and 800 rpm. The reaction was quenched with aqueous saturated NaHCO_3_ (250 µL) and extracted with EtOAc (2 × 250 µL). The combined organic layers were dried over Na_2_SO_4_ and subsequently analyzed on GC. The two amine enantiomers were separated on a chiral stationary phase (see chromatograms in Additional file 1: Figures S3, S4.) using a β-cyclodextrin capillary column (CP-ChiraSil-DEX CB; 30 m × 250 µm × 0.25 µm) with an injection and detection temperature of 250 °C (temperature program: 120 °C, 5 °C min^−1^ to 180 °C, hold 2 min).

## Additional file

**Additional file 1.** Additional table and figures.
