# Supplementary material for: In vivo plug-and-play: a modular multi-enzyme single-cell catalyst for the asymmetric amination of ketoacids and ketones
Source: Microb Cell Fact. 2017 Jul 28;16:132. doi: 10.1186/s12934-017-0750-5 (PMC5534079; doi:10.1186/s12934-017-0750-5)
Supplement: Supplementary file 1 — Additional file 1. Additional table and figures. [file 12934_2017_750_MOESM1_ESM.pdf]

Table S1. List of Oligonucleotides used in this study

**Oligonucleotides**	**Sequence 5'→3'**
ald$_{Bs}$_RBS_fw	CCGGAATTC*GAAAGGAGGCCCTTCAG***ATG**ATCATAGGGGTTCCTAA
ald$_{Bs}$_rv	CCGGGAATTC**TTA**AGCACCCGCCACAGATG
ilvE$_{Ec}$_RBS_fw	CCGGCCGGCCGGAATTC*AAGGAGATATAGAT***ATG**ACCACGAAGAAAGCTGA
ilvE$_{Ec}$_rv	CCGGCCGGCCGGCCGAATTC**TTA**TTGATTAACTTGATCTA
ilvE$_{Sm}$_RBS_fw	CCCCCCGCCGGCGAATTC*AAGGAGATATAGAT***ATG**ACAGTAGATTTAGATTG
ilvE$_{Sm}$_rv	CCGGCCGAATTC**TTA**GTCATCAACTTTATATA
ta$_{Cv}$_fw	TATATGAATTC**ATG**CAGAAACAGCGTACC
ta$_{Cv}$_rv	TATAGTCGAC**TTA**GGCCAGACCACGTG
*fdh*_RBS_fw	CCGGGAGCTC*AAGGAGATATAGAT***ATG**AAAATCGTTCTCGTTTT
*fdh*_rv	CCGGTCTAGA**TTA**TGCGACCTTTTTGTCAT
*gdh*_RBS_fw	CCGGGAGCTC*AAGGAGATATAGAT***ATG**TATAAAGATCTGGAAGG
*gdh*_rv	CCGGTCTAGA**TTA**TTAGCCACGACCCGCCT
*ptdh*_RBS_fw	CCGGGAGCTC*AAGGAGATATAGAT***ATG**CTGCCGAAACTGGTCAT
*ptdh*_rv	CCGGTCTAGA**TTA**GTCAGCCGCAGGGTTGGCTT
pBAD28_fw	CTGACGCTTTTTATCGCAAC
pBAD28_rv	CAGACCGCTTCTGCGTTCTG
GA_*ald*_RBS_*Eco*RI_fw	GCCGGGAATTC*AAGGAGATATAGAT***ATG**ATCATAGGGGTTCCTAA
GA_*ald*_rv	GCCCGAGCTC**TTA**AGCACCCGCCACAGATG
GA_*avtA*_RBS_fw	CCGGGAGCTC*AAGGAGATATAGAT***ATG**ACATTCTCCCTTTTTGG
GA_*avtA*_rv	CCGGGGATCC**TTA**GTGACTTTCAGCCCAGG

GA_*kan_EcoR*I_fw	ATTTCACACAGGAAACAGACCATGGTTGAGCGATTGTGTAGGCTG
GA_*kan*_rv	TGAATATCCTCCTTAGTTCCTTCCGGGGATCCGTCGACCT
GA_trc_fw	GGAACTAAGGAGGATATTCAGACATCATAACGGTTCTGGC
GA_trc_ rv	TATCTATATCTCCTTGGATCCCCGGGTACCGAGCTCGAATT
GA_*ald*_RBS_fw	GGATCC*AAGGAGATATAGAT***ATG**ATCATAGGGGTTCCTAA
GA_*ald_EcoR*I_rv	CCGGGTACCGAGCTCGAATTATGGGAATTAGCCATGGTCC**TTA**AGCACCCGCCACAGATGATTC
GA_*rocG*_RBS_fw	GGATCC*AAGGAGATATAGAT***ATG**AGCGCGAAACAGGTGAG
GA_*rocG_EcoR*I_rv	CCGGGTACCGAGCTCGAATTATGGGAATTAGCCATGGTCC**TTA**CACCCAGCCACGAAAAC
HS_*araCB*_ fw	ACTCTCTACTGTTTCTCCATACCCGTTTTTTTGGATGGAGTGAAACG**ATG**GTGTAGGCTGGAGCTGCTTC
HS_*araD*_rv	GTTTGATTGGCTGTGGTTTTATACAGTCA**TTA**CTGCCCGTAATATGCCTTATGGGAATTAGCCATGGTCC
*araC*_fw	ATTAGCGGATCCTACCTGAC
*polB*_rv	GCCTGGTTTCGTTTGATTGG
Kt_rv	GGCTGGGTGTGGCGGACCGC

Enzymatic restriction sites are underlined, homologous sequences of *E. coli* genome are scored, ribosomal binding sites are shown in italics, stop and start codons are highlighted in bold. Abbreviations: RBS: ribosomal binding site; GA: Gibson assembly, HS: homologous sequences

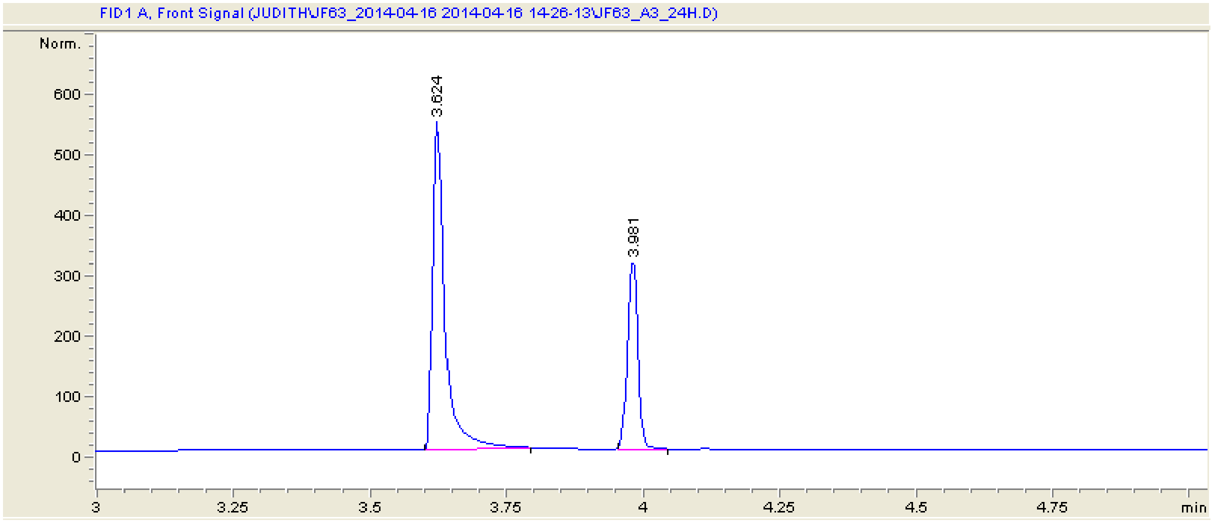

Figure S1. GC-chromatogram for determination of conversion in the reductive amination of 4-phenyl-2-butanone catalyzed by single-cell catalyst 6. The amine product was assigned by comparison of elution orders with commercially available reference material.

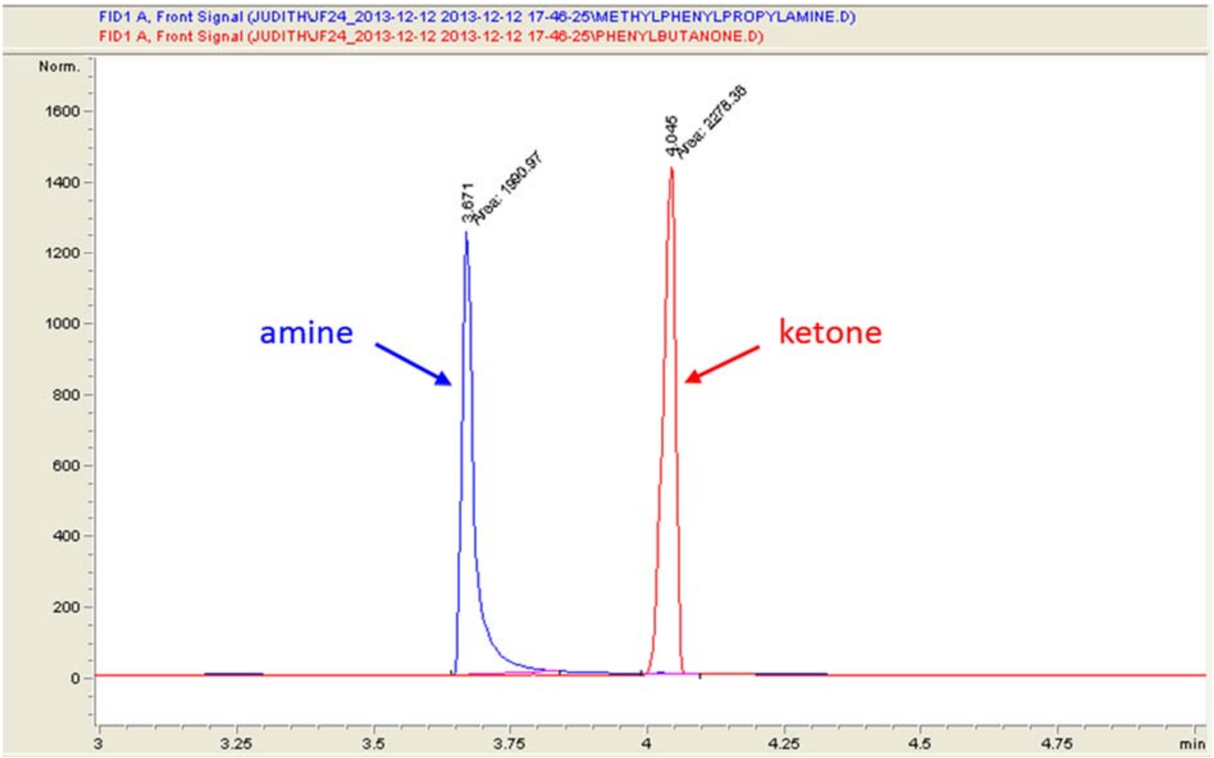

Figure S2. GC-chromatogram of reference compounds 4-phenyl-2-butanone and 1-methyl-3-phenylpropylamine.

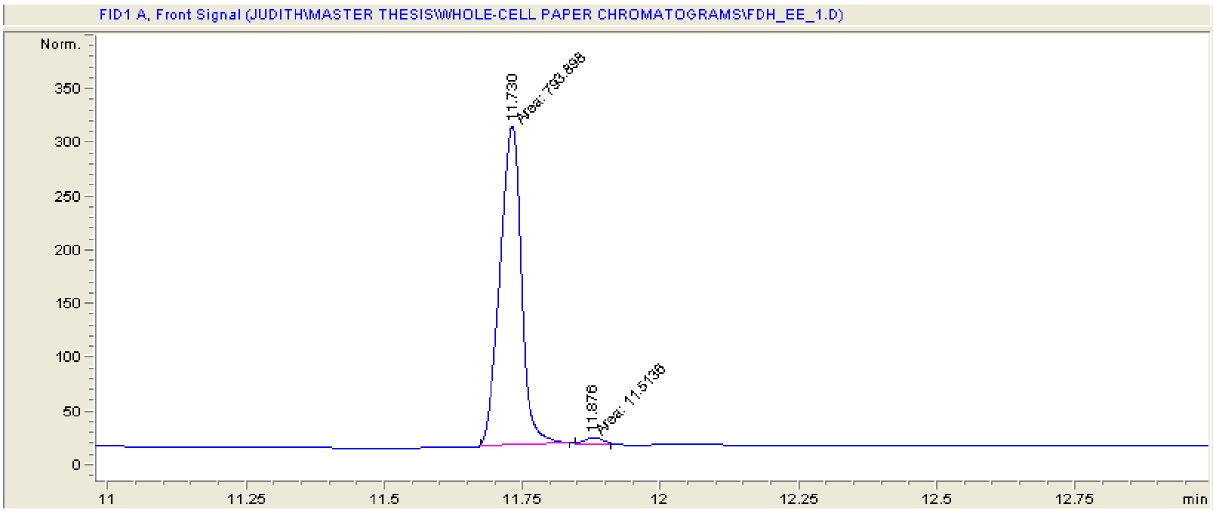

Figure S3. GC-chromatogram for determination of *enantiomeric excess* in the reductive amination of 4-phenyl-2-butanone catalyzed by single-cell catalyst 6. The absolute configuration was assigned by comparison of elution orders with commercially available optically pure amines.

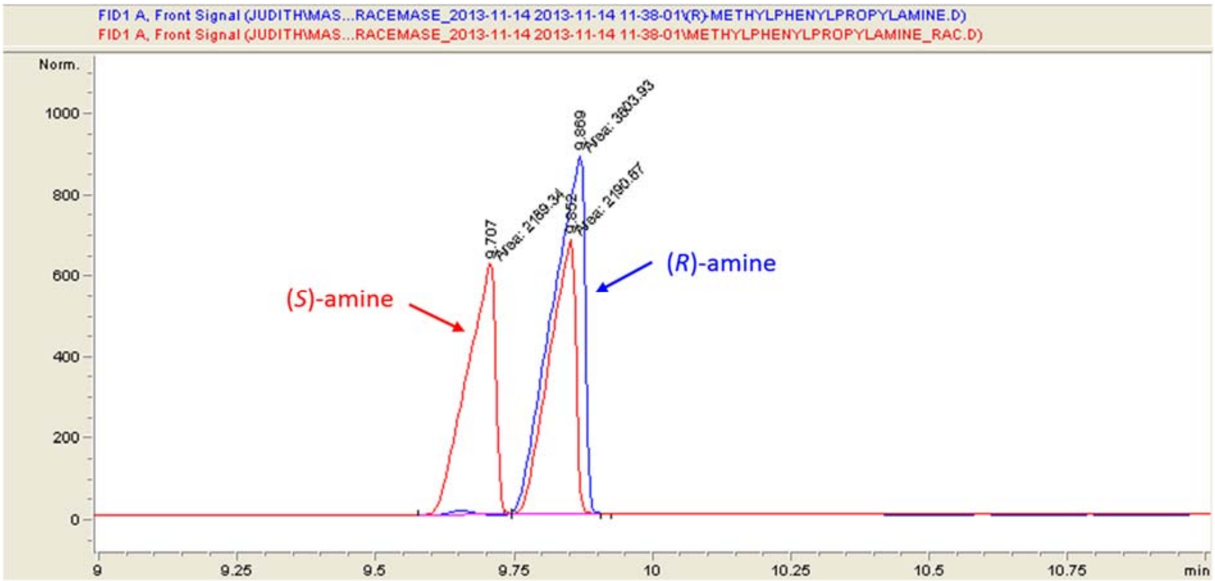

Figure S4. GC-chromatogram of racemic and optically pure reference compound (*R*)-1-methyl-3-phenylpropylamine.
